# Sheet-on-sheet fixed target data collection devices for serial crystallography at synchrotron and XFEL sources

**DOI:** 10.1107/S1600576724008914

**Published:** 2024-10-16

**Authors:** R. Bruce Doak, Robert L. Shoeman, Alexander Gorel, Stanisław Niziński, Thomas R.M. Barends, Ilme Schlichting

**Affiliations:** a Department of Biomolecular Mechanisms Max Planck Institute for Medical Research Jahnstrasse 29 Heidelberg69120 Germany; SLAC National Accelerator Laboratory, Menlo Park, USA

**Keywords:** sheet-on-sheet chips, serial crystallography, fixed target sample delivery, room-temperature crystallography, SFX, SSX

## Abstract

Serial crystallography (SX) efficiently distributes over many crystals the radiation dose absorbed during diffraction data acquisition, enabling structure determination of samples at ambient temperature. SX relies on the rapid and reliable replacement of X-ray-exposed crystals with fresh crystals at a rate commensurate with the data acquisition rate. ‘Solid supports’, also known as ‘fixed targets’ or ‘chips’, offer one approach. These are microscopically thin solid panes into or onto which crystals are deposited to be individually interrogated by an X-ray beam. Solid supports are generally patterned using photolithography methods to produce a regular array of features that trap single crystals. A simpler and less expensive alternative is to merely sandwich the microcrystals between two unpatterned X-ray-transparent polymer sheets. Known as sheet-on-sheet (SOS) chips, these offer significantly more versatility. SOS chips place no constraint on the size or size distribution of the microcrystals or their growth conditions. Crystals ranging from true nanocrystals up to microcrystals can be investigated, as can crystals grown in media ranging from low viscosity (aqueous solution) up to high viscosity (such as lipidic cubic phase). Here, we describe our two SOS devices. The first is a compact and lightweight version designed specifically for synchrotron use. It incorporates a standard SPINE-type magnetic base for mounting on a conventional macromolecular crystallography goniometer. The second and larger chip is intended for both X-ray free-electron laser and synchrotron use and is fully compatible with the fast-scanning *XY*-raster stages developed for data collection with patterned chips.

## Abstract 2

Fixed targets (‘chips’) offer efficient, high-throughput microcrystal delivery for serial crystallography at synchrotrons and X-ray free-electron lasers (XFELs). Within this family, sheet-on-sheet (SOS) chips offer noteworthy advantages in cost, adaptability, universality and ease of crystal loading. We describe our latest generation of SOS devices, which are now in active use at both synchrotrons and XFELs.

## 1. Introduction

In X-ray scattering studies of biological samples, damage to the sample by the X-ray beam itself is always a concern (Holton, 2009 ▸; Garman & Weik, 2023 ▸). For X-ray free-electron laser (XFEL) studies, this damage can be extreme: after the diffraction image has formed, the material at the X-ray beam focal spot simply disintegrates. This ‘diffraction before destruction’ (Chapman *et al.*, 2014 ▸) requires that XFEL diffraction data be collected serially, with each XFEL pulse exposing a different crystal (or part thereof) far away from sample regions previously exposed (Barends *et al.*, 2022 ▸). Serial crystallography (SX) is mandatory for XFEL measurements, but can also be useful in synchrotron X-ray studies, for example to study room-temperature protein dynamics (Thorne, 2023 ▸). Synchrotron SX is of particular interest at the ‘diffraction-limited’ high-brightness facilities that have emerged in recent years (Raimondi *et al.*, 2023 ▸; Robert *et al.*, 2023 ▸).

A wide variety of sample presentation methods has been developed for serial crystallography (Grunbein & Kovacs, 2019 ▸; Martiel *et al.*, 2019 ▸). Relevant to this paper is the category of ‘solid supports’ or ‘fixed targets’. These are microscopically thin solid panes into or onto which crystals are deposited to be individually interrogated by an X-ray beam (Zarrine-Afsar *et al.*, 2011 ▸; Mueller *et al.*, 2015 ▸; Sherrell *et al.*, 2015 ▸, 2022 ▸; Roedig *et al.*, 2015 ▸; Owen *et al.*, 2017 ▸). Silicon is often employed as the support material (Zarrine-Afsar *et al.*, 2011 ▸; Roedig *et al.*, 2015 ▸), although not exclusively, since other support materials may offer specific advantages such as transparency (Carrillo *et al.*, 2023 ▸) and/or reduced X-ray background [see supporting information for Doak *et al.*(2018 ▸): https://doi.org/10.1107/S2059798318011634/tz5098sup1.pdf]. The biocrystallography community has universally adopted the designation ‘chip’ for all such fixed target devices. Chips can be patterned with photolithography to produce a regular array of either blind recesses or funnel-shaped through-holes into which crystals can ‘self-localize’ as a crystal-containing solution is deposited onto the pane and excess solution blotted or suctioned away. To collect diffraction data, such a chip is rastered orthogonally through the X-ray beam. A periodic array of self-localization sites clearly requires stepwise rastering from site to site, pausing at each site for an X-ray exposure. There are several other general constraints (Barends *et al.*, 2022 ▸): The plane of the support must remain coincident with the focal plane of the X-ray beam and perpendicular to the X-ray beam direction. The X-ray beam must be centred accurately on a well or through-funnel for each exposure [see for example the discussion in the paper by Gotthard *et al.* (2024 ▸)]. The grid points must be sufficiently separated from one another that no exposure encounters damage propagating from a previous exposure elsewhere on the chip. Each well or funnel should ideally contain one and only one crystal. The loading process should consume a minimum of sample solution; in particular, very few crystals should be deposited outside of the localization sites and crystals should not be blotted or sucked away as excess solution is removed during sample loading. The sample must be protected from desiccation during loading and probing. If these criteria are met, chip-based measurements can greatly reduce sample consumption relative to that of a liquid jet. The necessary techniques and equipment have been developed (Horrell *et al.*, 2021 ▸), and for crystals that are 10 µm and larger in size this efficient use of sample appears to have been realized. For smaller crystals difficulties remain (Carrillo *et al.*, 2023 ▸), notably that such crystals may not be trapped in the self-localization sites or may simply fail to self-localize.

Nevertheless, sample loading onto patterned chips remains challenging. Patterned chips are also expensive, and a selection of pattern types may be needed to accommodate different samples. The sheet-on-sheet (SOS) sandwich removes many of these constraints (Doak *et al.*, 2018 ▸). The SOS device falls within – or perhaps ‘just outside’ – the solid support category of sample presentation methods. In essence, SOS dispenses with a patterned solid support pane and simply sandwiches a thin layer of crystal slurry (typically 20 µm thick) directly between two transparent polymer films (each typically 2–6 µm thick) to fill the entire window of the chip. The SOS ‘chip’ then does not actually involve a true ‘chip’ but rather only simple and inexpensive polymer sheets. Crystals position themselves more or less at random within the SOS slurry layer. The areal crystal density within the layer is chosen relative to the X-ray footprint and scan speed such that single-crystal diffraction dominates. The transparency of the polymer films allows fast visual control of sample spreading, crystal density and crystal slurry thickness.

SOS is basically a two-dimensional analogue of sample delivery by microscopic jet, with mechanical rastering rather than jet flow delivering pristine sample for each serial X-ray exposure. Thus the demands of maintaining a stable jet flow are avoided. Conventional serial X-ray exposures on a jet constitute a simple linear scan, with the only relevant ‘scan’ factors being the jet speed, the speed at which radiation damage progresses along the jet and the repetition rate of the X-ray exposures. By suitable choice of jet speed and X-ray pulse rate, up-stream diffusion of radicals into crystals yet to be probed can generally be obviated. In a ‘back-and-forth’ raster scan across an SOS chip, similar parameters enter but, since the scan necessarily returns periodically to the proximity of previously exposed regions, the number of relevant scan factors is increased. In particular, the spacing of X-ray exposures within a scan line may need to be different from the spacing between adjacent scan lines.

Since the crystal locations on an SOS chip are neither ordered nor pre-defined, SOS raster scans need not be point by point. Rather, line scans can be made while rastering the chip back and forth at essentially constant speed, while recording X-ray exposures ‘on the fly’. For XFEL exposures this approach is clearly viable, given that the scan displacement is sub-picometre on the femtosecond timescale of the XFEL pulse. The same is not true during the finite exposure of a synchrotron measurement, which however is necessarily bounded by the extent of the X-ray focal spot in the scan direction. This is typically only a few micrometres at a high-brightness synchrotron. A suitable scan speed and exposure time can then generally be chosen to yield usable diffraction for each exposure, in which case ‘on-the-fly’ SOS measurements are also possible at a synchrotron.

The only SOS consumables are the polymer films. The cost of these is minimal, so the financial outlay is mostly in the up-front cost of fabricating chip holders, mounts and loading apparatus. If machined from metal, these can generally be reused indefinitely. Lithographically patterned chips have similar up-front costs for mounts and loading apparatus, but the chips themselves can be very expensive and are often quite fragile and subject to breakage. They can sometimes be cleaned and reused, but never indefinitely. Moreover, if a range of grid sizes or well/funnel dimensions are needed, the costs increase proportionally. An SOS chip, having no fixed grid, avoids these complications. SOS scan parameters are set as desired for each scan and re-adjusted at will. Since they require no blotting or suction, SOS chips are straightforward to load with crystal solutions of a wide variety of compositions and viscosity, a major advantage relative to patterned chips.

**Figure 1:**
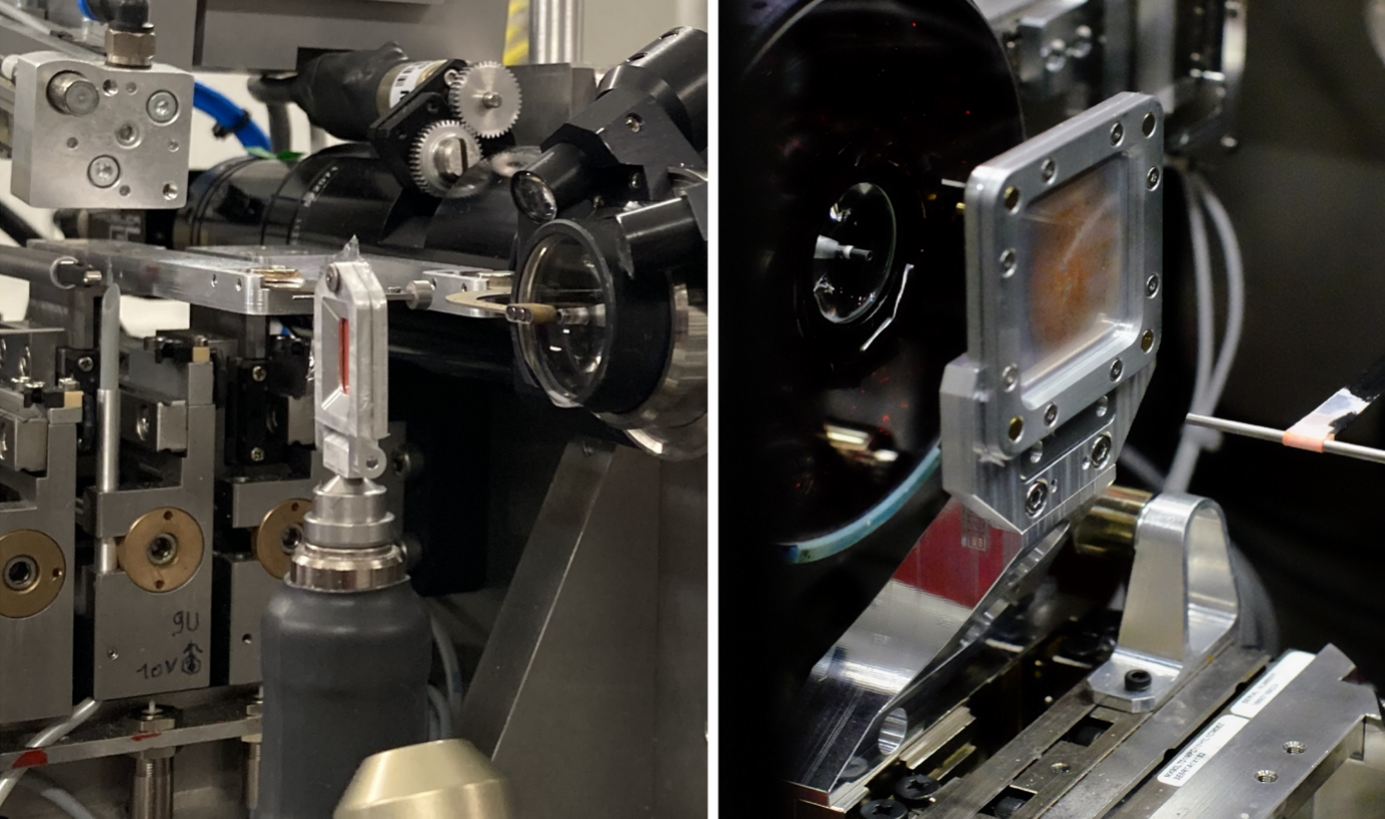
SOS sample presentation devices described in this paper. In both photographs, red-coloured microcrystals are mounted between two sheets of 2.5 µm-thick Etnom foil. The chips shown were machined from aluminium, which yields smooth sealing surfaces and a mechanically robust chip. However, 3D printing from PLA, ABS *etc.* is also an option. Left: the smaller SOSOS incorporates a standard magnetic-button mount compatible with most MX goniometers. It is shown mounted on the goniometer at the ID23-2 endstation of the ESRF synchrotron, Grenoble, France; the detector is off to the left. Right: the larger SOS chip employs magnetic attraction, not just of the loaded chip into a generic mounting cradle but also of the two frame halves to one another for ease assembly during sample loading. The SOS chip is shown positioned for data collection at the Cristallina-MX endstation of the SwissFEL X-ray Free-Electron Laser at the Paul Scherrer Institut, Villigen, Switzerland (see https://www.psi.ch/en/swissfel/cristallina/crmx-fixed-targets). In this view, the detector is off to the right. The SOS chip is placed into its mounting cradle from the detector side. By fabrication of an appropriate adaptor bracket, like that in the photograph, the generic cradle with its chip is readily attached to a fast-scanning stage.

Three major challenges in using SOS chips are spreading the sample layer uniformly and thinly over the film, avoiding the presence of wrinkles in the assembled sandwich, and avoiding adverse crystal settling during data collection. We have addressed these challenges by careful design refined through extensive testing of the SOS frames, the loading apparatus, the loading protocol and the scan parameters. Photographs of the two new SOS devices are shown in Fig. 1 ▸. The smaller of these has a 5 × 10 mm window. It incorporates a standard SPINE-type magnetic pin base, allowing ‘drop-in’ installation on most synchrotron macromolecular crystallography (MX) beamline goniometers. We refer to this as the SOSOS chip (‘SOS on SPINE’). Our second device is larger, with a 30 × 30 mm open scan window. To distinguish it from the SOSOS chip, we generally refer to it as the SOS chip (although we employ the same name as a general descriptor for both types). Both devices have been tested extensively in diffraction data collection at various XFEL and synchrotron facilities and by several different research groups. They offer a fast, efficient, simple and inexpensive means of collecting serial MX X-ray data.

## 2. Results and discussion

Our original SOS paper (Doak *et al.*, 2018 ▸) introduced the SOS methodology but described only the makeshift apparatus used in our seminal *ad hoc* measurements at SACLA. Some limited information on our subsequently developed SOS chips was provided in the supporting information of that seminal paper, but the SOSOS chips were not described at all. The present publication supplies the detailed information and understanding needed to meaningfully employ and even to fabricate both versions. Complete STP CAD files of the SOS components are included in the supporting information. Since both the SOSOS and SOS chips have reached a mature state, this paper constitutes a definitive reference, both for our own pending publications as well as for those already appearing elsewhere by other groups. Users then need not incorporate such information into their own publications (Grieco *et al.*, 2024 ▸), and the provenance of the devices will be unambiguous (Stubbs *et al.*, 2024 ▸).

The small sample volume and its ease of use make the SOSOS chip particularly useful for sample testing and collection of reference datasets at a synchrotron (Grieco *et al.*, 2024 ▸; Stubbs *et al.*, 2024 ▸). These chips require no adaptation if the instrument of interest incorporates a standard SPINE magnetic button. Mounting of the SOS chip at an XFEL or endstation does in general require a custom-made bracket, but our generic SOS mounting cradle can often be modified or augmented for this. Both the SOSOS and the SOS chips mount magnetically, allowing for extremely easy, rapid and reproducible insertion and removal.

Both types of chip provide automatic wrinkle-free mounting of the polymer films by forcing a slight stretching of the film over a raised boss or shoulder as one-half of the frame is clamped against the other. The clamping also creates the seal between the two films. In the absence of the designed-in film stretching, achieving wrinkle-free films requires significant skill, practice and luck. With the designed-in stretching, it becomes routine. The magnetic attachment and self-aligning nature of the SOS chip halves simplifies and greatly accelerates sample loading, which in turn reduces the danger of sample desiccation. Sample loading and assembly are described in detail for both chips below and in the supporting information.

### 2.1. Chip design

#### 2.1.1. SOSOS – sheet-on-sheet on SPINE

**Figure 2:**
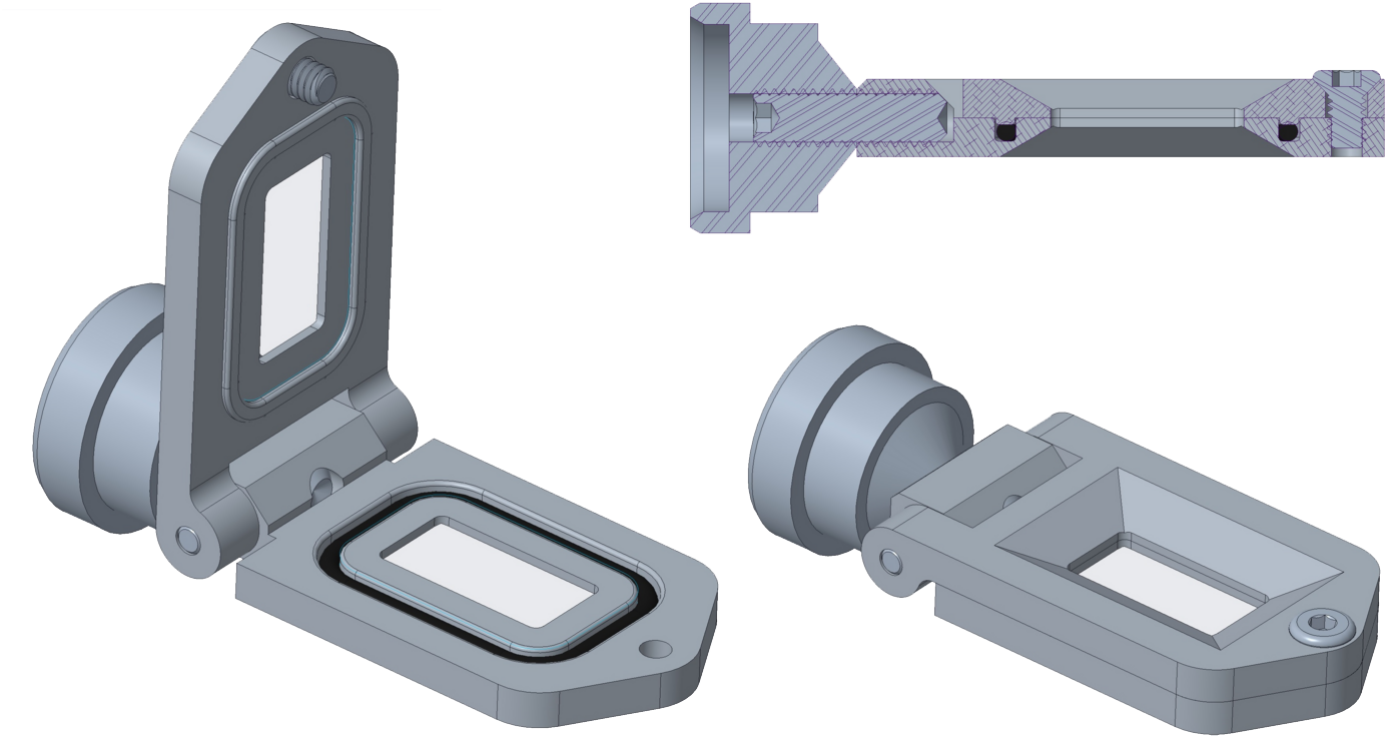
CAD screenshots of the SOSOS device. Views are shown with the hinged frame section opened for loading (left) and clamped closed for measurements (bottom right). In the clamped configuration, as seen in the sagittal cross-sectional view (upper right), a raised boss compresses the two SOS polymer films (not shown) against each other and against an O-ring to seal the sample between the films and also stretch out wrinkles in the films. The open window is 10 mm long and 5 mm wide. A detailed description of the constituent components is given in Fig. S1 and Table S1.

To accommodate standard MX goniometer mounting, the SOSOS was intentionally designed to be compact and lightweight, even when the chip is fabricated from aluminium. Images of the SOSOS, taken directly from 3D CREO (https://www.ptc.com/en/products/creo) design drawings of the chip, are shown in Fig. 2 ▸. The design incorporates a commercial magnetic pin-base MX goniometer mount. Alternative mounts are possible. At ESRF endstation ID29, for example, the SPINE goniometer base was replaced with a proprietary base unique to ID-29 (Grieco *et al.*, 2024 ▸; Stubbs *et al.*, 2024 ▸).

To simplify loading, the two halves of the SOSOS are attached by means of a hinge. This allows the ‘cover’ to be flipped open relative to the ‘base’ for sample loading, then flipped shut and sealed by tightening a single screw. The window of the SOSOS chip is 10 mm long along the axis of the goniometer and 5 mm wide in the lateral dimension, and extends from 16.6 to 26.6 mm from the standard SPINE reference surface. The internal corners of the window partially shadow the detector in 0.5 mm-wide regions near the window edges. Provided the raster scan is restricted to the 4 × 9 mm central area of the window, scattered X-rays emerge unobstructed at angles of at least 60° to the X-ray beam

As seen in a cross-sectional view (Fig. 2 ▸, upper right), clamping the SOSOS chip shut compresses a boss against an M13x1 O-ring in an O-ring groove. The intent is twofold: (1) the two sheets, captured between the boss and the compressed O-ring, are forced tightly together to form a hermetic seal; (2) the 0.35 mm-high boss slightly stretches the two films during clamping, serving to smooth out wrinkles in the films. Once clamped, the two films lie exactly on the axis of the magnetic button, so it makes no difference which of the two frame halves faces the detector. Nonetheless, it is useful to establish and maintain a convention (*e.g.* with the head of the clamping screw head always facing the detector), since knowing the exact film orientation during the scan is important if diffraction patterns are to be analysed post-measurement as a function of the position on the chip (Gorel, 2024 ▸).

**Figure 3:**
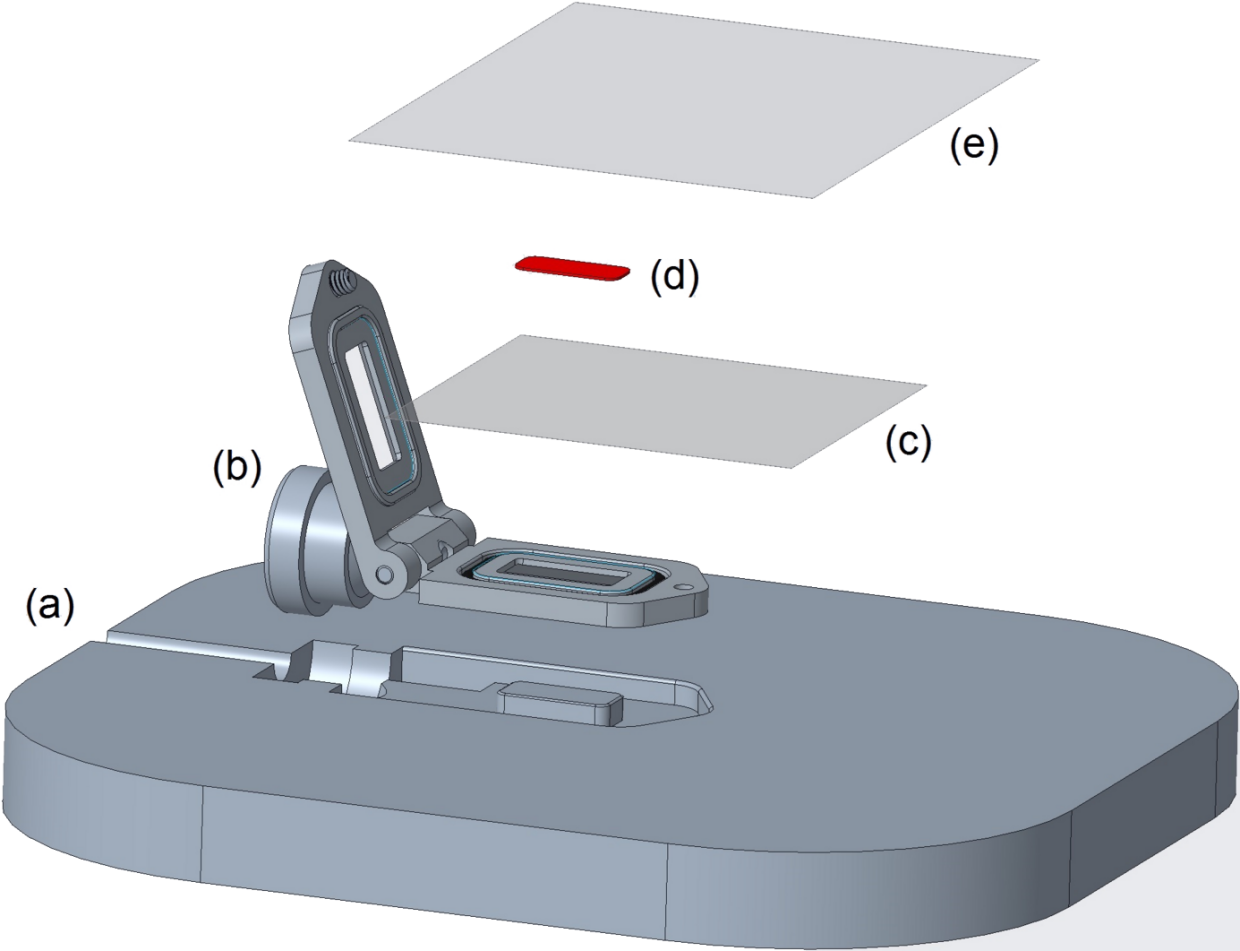
‘Exploded’ diagram depicting SOSOS chip loading. (*a*) Loading plate with form-fitted recess for the chip. (*b*) SOSOS chip with hinged cover flipped open. (*c*) Lower polymer sheet. (*d*) Sample, spread on the lower sheet. (*e*) Upper polymer sheet.

To load the SOSOS chip, it is placed in a form-fitted recess in a 3D-printed loading block, as indicated in the exploded view of Fig. 3 ▸, and the frame is flipped opened. A small amount of viscous grease (vacuum grease, Superlube, or similar) is spread on the loading plate just outside the recess. A roughly 30 × 30 mm piece of the desired film is carefully placed over the SOSOS chip and spread to remove wrinkles, tacking the film into place in the grease and taking care not to pull any grease into the window region. A small volume of sample slurry, typically 5 µl, is then pipetted onto the film and spread as thinly and uniformly as possible across the central 9 × 4 mm area within the open window. We typically add 1–2% hy­droxy­ethyl cellulose to an aqueous crystal storage solution (see Section 2.2). A central pedestal in the loading plate projects into the SOSOS window area to provide a solid support under the films during sample loading (the top of the pedestal is flush with the sagittal plane of the device and with the upper surface of the loading plate). An additional small amount of viscous tacking material is then spread on the outer periphery of the loading plate, being careful not to disturb the first film and sample. A larger piece of film (*ca* 50 × 40 mm) is added to the stack, tacked down into the grease and manipulated carefully to remove as many wrinkles as possible. The hinged frame is lowered into its closed position and the clamping screw inserted and tightened just far enough to clamp the films against the O-ring on the hinge side. The sample can spread through capillary action alone, but we prefer using a custom-made hand press at this juncture to carefully distribute the sample into a thin uniform layer between the two polymer films (see the supporting information). After pressing, any remaining wrinkles are again removed insofar as is possible by pulling gently against the clamped side of the films. The clamping screw is then tightened fully. Remaining minor wrinkles should disappear as the screw is tightened. The excess film material is cut away by running a scalpel blade around the periphery of the chip. The chip is then removed from the loading platform and mounted on the goniometer.

#### 2.1.2. SOS – large sheet-on-sheet chips

Since they are specifically designed for synchrotron goniometer use, SOSOS chips offer only a modest number of X-ray of exposures per chip, generally well under 90 000 even if damage spreading allows a minimal 20 µm spacing both within and between line scans, and so delivering perhaps only 20 000 indexed diffraction patterns. In many instances, a significantly higher yield of indexed lattices per chip is desired.

**Figure 4:**
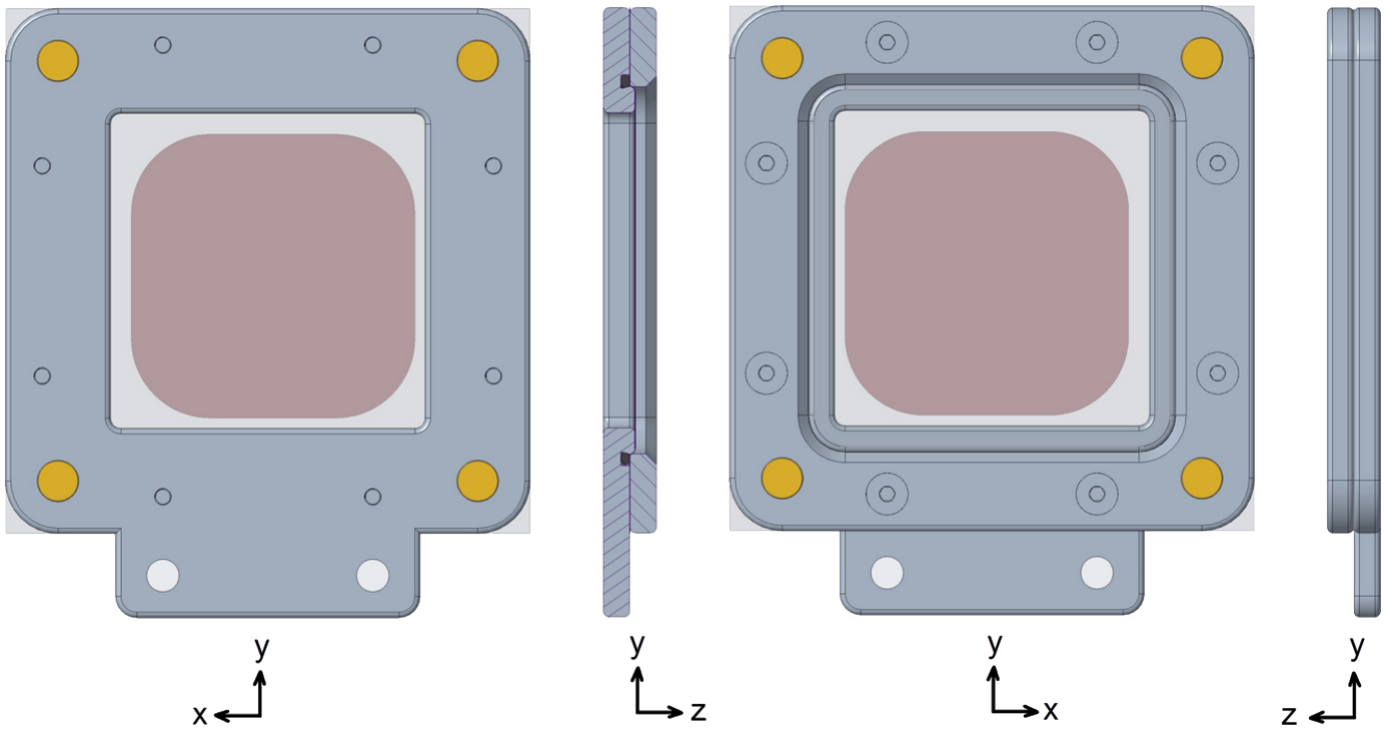
Screenshots from 3D CAD drawings of the larger SOS chip. The through-window is 30 × 30 mm. The chip is asymmetrical and the standard mounting orientation relative to the X-ray beam is as indicated (the *y* axis is directed vertically upwards and the *z* axis along the X-ray beam). The chip fits into its cradle only in this orientation, and this produces no shadowing of the detector by frame edges from any point within the through-window. The constituent components are listed in Fig. S4 and Table S2.

For that reason, we designed a second, larger SOS chip with a 30 × 30 mm window facing the X-ray source, as illustrated in Fig. 4 ▸. Despite the large size of the SOS chip, its mass is still under 23 g fully loaded, even when fabricated from aluminium. The SOS chip has no hinged joint; rather the base plate and cover plate separate fully. Small disc magnets are press-fitted or glued into cylindrical recesses in the corners of these two plates to hold the two plates firmly together when they are brought into near proximity. This greatly simplifies assembly of the chip during loading. The stacking of the SOS components follows that of the SOSOS. A small volume of sample slurry, typically 10–15 µl [25–30 for viscous lipidic cubic phase (LCP)], is then pipetted in small evenly spaced droplets across the central area within the open window. As with the SOSOS loading, we typically add 1–2% hy­droxy­ethyl cellulose to the aqueous crystal storage solution (see Section 2.2). The sample layer is sealed between the two SOS films by clamping them together against an M42x1 O-ring. To ensure fully adequate compression of the O-ring, eight screw holes about the frame periphery (tapped through-holes in the base plate, countersunk clearance holes in the cover) accept M2x0.4 flat-head screws. The screws are tightened firmly, working in a symmetric pattern about the periphery to compress the O-ring uniformly. Overtightening the screws can strip the small M2 threaded holes.

**Figure 5:**
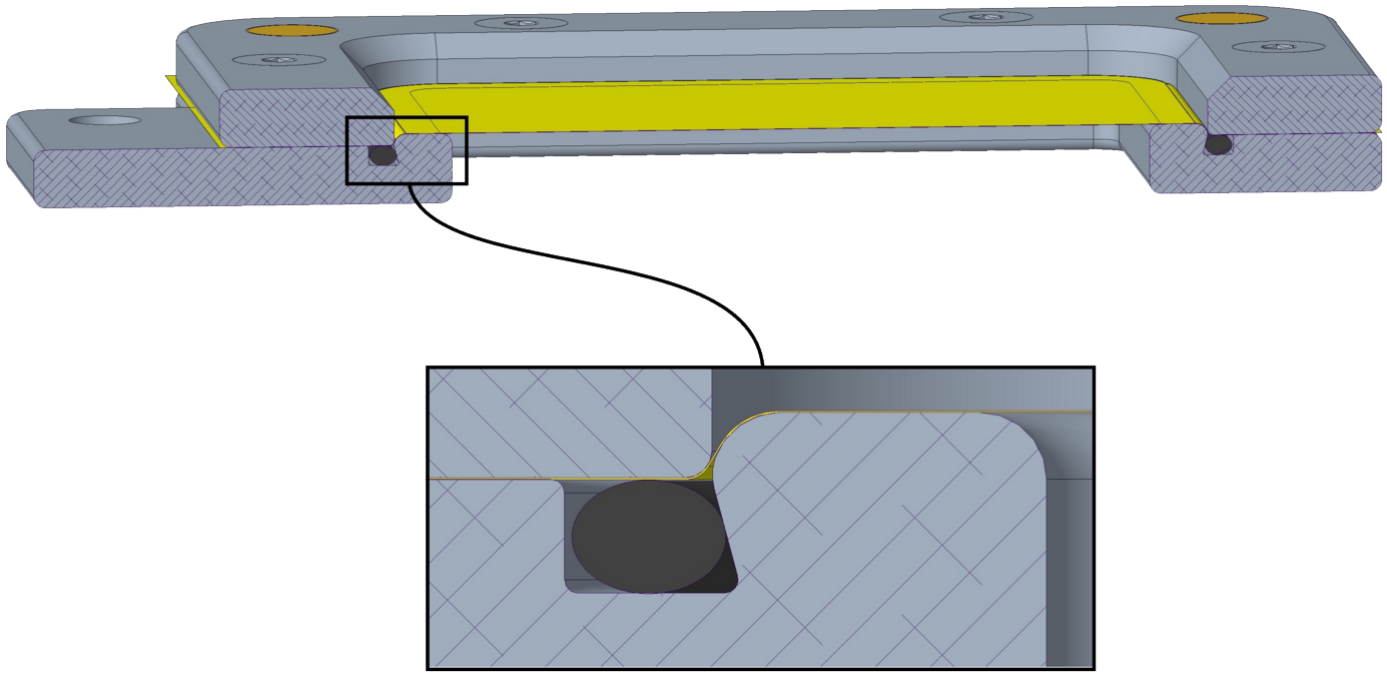
The sample layer is sealed between two polymer films by compressing the two films together against an underlying O-ring. As the O-ring compresses, the films are pulled laterally outwards over the rounded corner of a raised boss surrounding the window in the base plate. The surface of this boss projects 0.50 mm into the opening in the cover plate. Stretching helps remove any residual wrinkles in the films. The cover and base plate are both 2.50 mm thick, the films typically 2–6 µm thick and the sample layer typically 20 µm thick.

The sealing geometry differs slightly from that of the SOSOS chip. As seen in Fig. 5 ▸, a 0.5 mm-high boss surrounds the 30 × 30 mm open window in the base plate and projects with tight clearance into the 35 × 35 mm opening in the cover plate. As the clamping screws are tightened, the films are stretched laterally outwards and downwards over the rounded periphery of the boss simultaneously with formation of the sheet-to-sheet seal. The O-ring groove of Fig. 5 ▸ is shown with a slanted inner wall that captures the O-ring. This construction is amenable to 3D printing, but if the parts are to be machined, an O-ring groove with simple parallel sides is easier to fabricate and also suffices. The 0.5 mm offset of the sample plane from the contact plane between the cover and the base should be noted when designing a mounting bracket, particularly if a shift in the scattering plane by this small amount might have experimental ramifications.

A generic mounting cradle for the chip, visible immediately below the SOS chip in the right-hand photograph of Fig. 1 ▸, incorporates two magnets to hold the chip in place via magnetic attraction. A small tab (8 × 29 mm with two M3 through-holes) was also included as part of the base plate for rigid screw attachment to the generic bracket, if needed. The two screws are rarely employed (the screw holes are seen to be empty in Fig. 1 ▸), but the tab also sets the orientation of the mounted chip by mating into a corresponding recess in the generic cradle. Thus, there is only a single unique orientation in which the chip can be mounted. In post-exposure analysis of the films, there is then no question as to which chip side was up, which was facing the X-ray source *etc*. Two additional screw holes allow the generic cradle to be screwed to a strut designed to fit the experimental geometry at hand, rather than fabricating a more complicated one-piece mount. This approach is shown in Fig. 1 ▸.

With the chip oriented relative to the X-ray beam as indicated in Figs. 1 ▸ and 4 ▸, X-rays scattered from anywhere within the 30 × 30 mm through-window can deflect by at least 60° with respect to the X-ray beam without any shadowing of the detector by the edges of the frame (if the frame were flipped relative to the X-ray beam, only the central 20 × 20 mm area of the window would meet this criterion).

**Figure 6:**
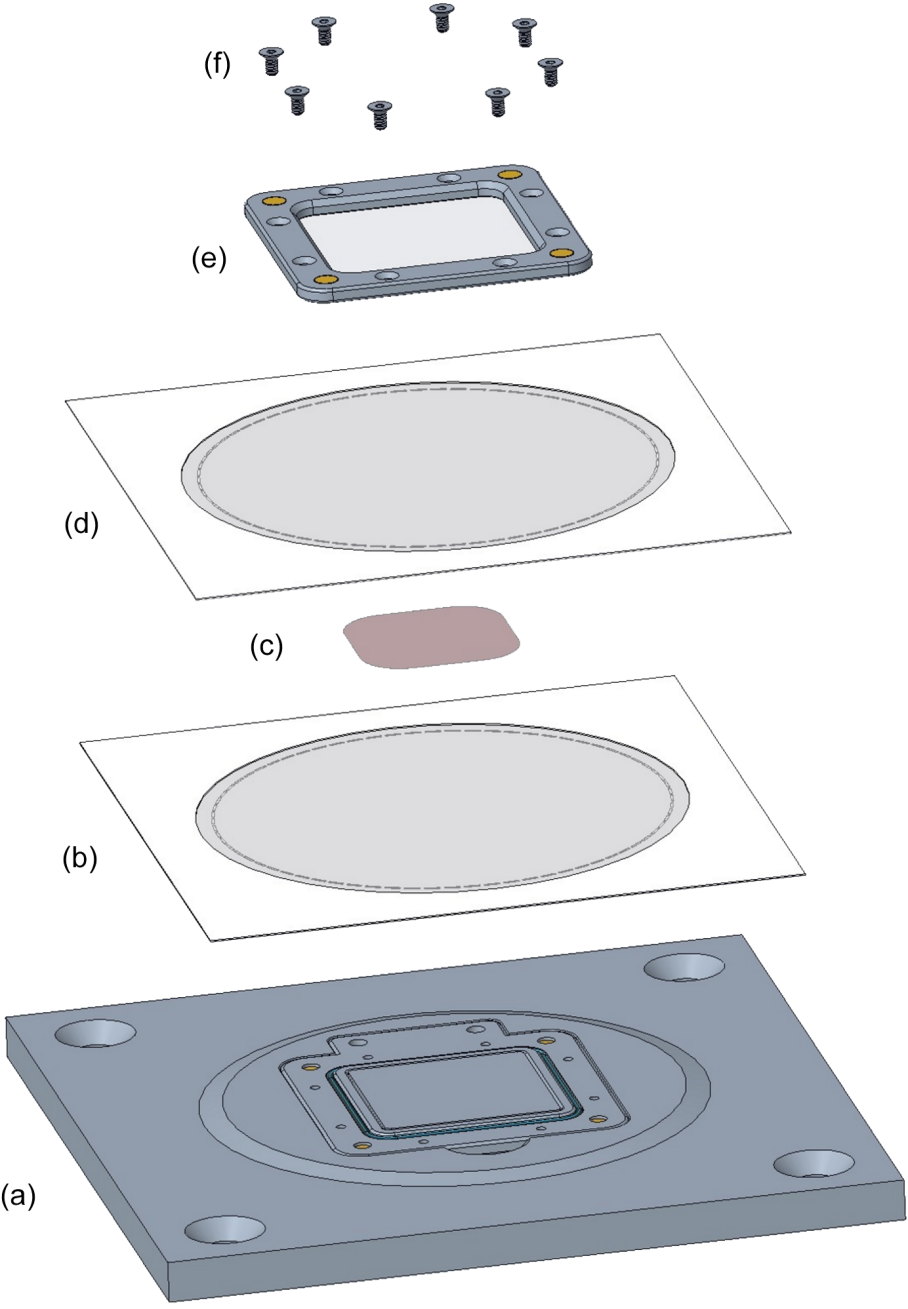
‘Exploded’ diagram depicting SOS chip loading. (*a*) The base plate of the chip is placed in a form-fitted recess in a slightly raised plateau on a loading plate. (*b*) A first film is placed over the base plate. (*c*) Sample slurry is spread over the film within the window region. (*d*) A second film is added and then (*e*) the cover plate. The baseplate and cover plate each contain four magnets, oriented to hold two plates together firmly but not overly tightly. If necessary, major wrinkles can be removed by tugging at the periphery of the films. (*f*) Eight clamping screws are then inserted and tightened, pulling the cover firmly against the base plate and forcing the two films together against the O-ring as it is compressed to seal the sample inside the films while also stretching out any remaining wrinkles. As with the SOSOS chip, a press can be used to decrease the thickness of the sample layer and increase its uniformity before the screws are fully tightened (see the supporting information).

As depicted schematically in Fig. 6 ▸, the SOS chip is loaded in a manner similar to that described for the SOSOS chip. The chip base is placed in a form-fitted cut-out in a loading plate. Four magnets mounted into the back of the loading plate hold the base half of the chip frame securely in the recess. The exposed surface of the frame is flush with the surface of the loading plate. A raised pedestal in the centre of the cut-out extends into the 30 × 30 mm window, such that its upper surface is flush with the surface of the 0.5 mm-high boss. The SOS loading platform was specifically designed to accommodate standard polymer films with a 3 inch-diameter perforation circle and encased in 4 × 4 inch paper frames (*e.g.* Chemplex). The paper frame promotes wrinkle-free mounting, ease of handling, improved cleanliness and reduced electrostatic charging. Alternatively, unframed polymer sheets can be tacked into place with a viscous grease, as done with the SOSOS chips. After the first film has been laid in place, the sample slurry spread over it and the second film positioned atop the sample layer, the cover plate can be carefully placed into position atop the stack, where it is held in place magnetically. We generally use a mechanical press at this juncture to both thin the sample layer and render it as uniform as possible (see the supporting information). Then the screws are tightened to compress the cover against the films and the O-ring, sealing the sample layer inside the films. Any residual small wrinkles will generally disappear as this is done. A scalpel blade is run around the periphery of the chip to cut away the outlying excess film material before the chip is removed from the loading plate.

### 2.2. Using the chips

When using the chips, the first matter of concern is to choose the material properties and thickness of the polymer film. Chemical resistance and tensile strength are not of major concern. Chemical composition and thickness are critical, however, as these influence both the X-ray scattering from the film (and thereby the X-ray background in a diffraction measurement) and the loss of water through the film (which can affect background, isomorphism and resolution). X-ray transparency can be calculated (see for example https://henke.lbl.gov/optical_constants/) or looked up (see for example Chemplex). Water loss through the film is readily characterized by a simple measurement of the mass of the loaded chip as a function of time. To do so, 20 µl of water is loaded between two polymer sheets (see below and the supporting information). The chip is placed on a balance and its mass recorded at intervals for several hours. Any balance of 25 g capacity and 1 mg resolution suffices, provided it holds its calibration to within a milligram or so over the course of the measurement. Since the mass of the entire assembled and loaded chip is monitored, any leakage out through the film seals is automatically included. Such measurements can be made with water or with actual sample or mother liquor to deliver the ‘working time’ for desired film materials, film thicknesses and samples.

We typically use 2.5–3 µm-thick Etnom sheets for crystals in aqueous solutions and 6 µm-thick Mylar sheets for crystals in LCP. The sheets can be used without further treatment (cleaning, chemical treatment *etc.*). Since water can and will evaporate through films of the above thicknesses, it is advisable to fill the chip immediately before data collection. Alternatively, the chips can be stored in a humidity chamber.

Apart from the polymer films, the next most important contribution to background scattering comes from the solvent or other material (*e.g.* LCP) in which the crystals are immersed. It is thus highly desirable to reduce the thickness of the crystalline slurry layer to the minimum possible. Ideally the slurry layer should fill a chosen area (often the full chip window) with a sample layer less than 20 µm thick (or the crystal thickness if larger). The requisite volume of sample is readily calculated and pipetted onto the chip film. Good starting volumes are 3–4 µl for the SOSOS chip and 10 µl for the SOS chip when using low-viscosity crystalline slurries. LCP samples need significantly higher volumes (20–30 µl for covering the entire SOS chip). The challenge is to spread the slurry uniformly into a thin layer of the desired thickness and covering the desired area. To this end, we usually dispense the crystalline slurry in small droplets across the entire chip area and then use a specially designed press for mechanical spreading (see the supporting information). We prefer not to rely only on the magnetic attraction between the two SOS frame halves for film stretching and sealing, but rather take the 2 min needed to insert and tighten eight M2 clamp screws around the periphery of that frame. Since the polymer films are transparent, the thickness of the crystalline slurry in the SOS chip can be measured with a microscope by focusing first on the top sheet and then on the lower sheet, and noting the displacement. Our layers of crystalline slurries are typically 20–30 µm thick (see Fig. S7 of the supporting information).

Perhaps surprisingly, the crystals in an SOSOS/SOS can ‘flow’. SOS X-ray exposures are inevitably made with the chip oriented vertically. Small and dense crystals can settle downwards through even very thin SOS channels between the films. The settling speed (and direction: buoyant crystals would ‘settle’ upwards) depends on the viscosity of the carrier liquid, the crystal size, and the relative densities of the crystals and the surrounding solution. Settling is often negligible but, if not, may influence the choice of scan procedures. For example, completing a scan as quickly as possible becomes even more important, and the storing of loaded SOS frames horizontally rather than vertically prior to mounting is advisable. (sup­porting information). To prevent sample settling yet allow leeway in adjusting the crystal slurry layer thickness, we often add 1.5–2% hy­droxy­ethyl cellulose to the mother liquor. This also improves wetting of the polymer films. We have not observed any negative effects, in particular in the level of background scattering, due to this addition of hy­droxy­ethyl cellulose.

We have used the SOS chips extensively, both for characterization of the chips and for actual scientific measurements. Considerable effort was devoted to determining how closely the SOS X-ray exposures can be spaced, both inter- and intra-line, while still avoiding X-ray damage issues (Gorel *et al.* in preparation; see the supporting information). Much of that testing was carried out at ESRF endstation ID29 and Cristallina-MX. We supplied those endstations with aluminium versions of both the SOSOS (ID29) and SOS (Cristallina MX) chips, the corresponding film mounting plates, the specially designed mounting brackets, instruction in loading and using the chips, and CAD drawings of all the components. Those chips and loading stations are now available for users (*e.g.* Grieco *et al.*, 2024 ▸; Stubbs *et al.*, 2024 ▸).

The large SOS chip has also been used at the ID29 SMX endstation, both to collect data for model systems and with *bona fide* samples. Excellent high-resolution data can be obtained (the statistics of a thaumatin dataset are given in Tables S4 and S5 of the supporting information). Elsewhere, measurements with the SOS chip have been made at the MFX endstation at LCLS, SLAC National Accelerator Laboratory, USA (data not shown), and at the Cristallina endstation at SwissFEL [data not shown, but see https://www.psi.ch/en/swissfel/cristallina/crmx-fixed-targets and Got­thard *et al.* (2024 ▸)].

## 3. Concluding remarks

SOS chips are unique in their cost effectiveness and in their ease and versatility of use with crystals of both soluble and membrane proteins, as grown in both low-viscosity (aqueous solutions) and high-viscosity media (LCP). They are not intended to replace lithographically patterned chips. In particular the latter have the great potential advantage of isolating crystals at the bottom of a well or hole, thereby mitigating propagation of X-ray beam damage from one exposure to the next and also serving to avoid premature photo-excitation in photo-initiated time-resolved measurements (Gotthard *et al.*, 2024 ▸). In SOS measurements, these detrimental processes can be alleviated only by increasing the spacing of X-ray exposures, and thereby decreasing the efficiency of sample consumption (see the supporting information).

Sample consumption is a matter of concern in all X-ray scattering measurements, the figure of merit being the mass of protein consumed to deliver the desired protein structure. Relevant to this metric are the mass of protein present per volume in the sample slurry of interest (after mixing, settling *etc.*), the volume of this slurry loaded onto the chip, the loss of protein due to blotting/suction or failure of self-localization during loading, the number of X-ray exposures that can be made in scanning a particular chip, the number of useful diffraction images that result (accounting for hit and indexing rates), and the number of indexed diffraction images required to a complete structure determination. This information is not routinely documented for patterned chips, although that may be changing. For example, the advent of transparent chips now allows self-localization of small crystals to be characterized accurately by optical microscopy (Carrillo *et al.*, 2023 ▸), whereby it was found that moderately sized crystals (25 µm) self-localize rather well but smaller crystals (5–10 µm) do not. Self-localization is not an issue with SOS chips, of course, nor is crystal size or crystal loss due to blotting or suction during loading. The volume of sample pipetted onto an SOS sheet can be exactly known, and so likewise the number of crystals on the film provided the density of crystals in the sample slurry is known or measured. It may be that SOS measurements are more sparing of sample than is often imagined.

## 4. Related literature

The following references are cited in the supporting information: Dickerson *et al.* (2024 ▸); Murshudov *et al.* (2011 ▸); Nass *et al.* (2016 ▸); Shoeman *et al.* (2023 ▸); Sorigue *et al.* (2021 ▸); White *et al.* (2012 ▸).

## Supplementary Material

STEP files. DOI: 10.1107/S1600576724008914/te5135sup2.zip

Supporting figures and tables. DOI: 10.1107/S1600576724008914/te5135sup1.pdf
