# Supplementary material for: Sheet-on-sheet fixed target data collection devices for serial crystallography at synchrotron and XFEL sources
Source: J Appl Crystallogr. 2024 Oct 16;57(Pt 6):1725–32. doi: 10.1107/S1600576724008914 (PMC11611291; doi:10.1107/S1600576724008914)
Supplement: Supplementary file 2 [file j-57-01725-sup1.pdf]

JOURNAL OF
APPLIED
CRYSTALLOGRAPHY

Volume 57 (2024)

Supporting information for article:

Sheet-on-sheet fixed target data collection devices for serial crystallography at synchrotron and XFEL sources

R. Bruce Doak, Robert L. Shoeman, Alexander Gorel, Stanisław Niziński, Thomas R.M. Barends and Ilme Schlichting

S1. SOSOS chip

The constituent components of the basic SOSOS chip are shown and labelled in the exploded view of **Figure S1**. Details are provided in the following **Supplementary Table 1**. With the base plate and cover machined from aluminium, the mass of the assembled device is 7.0 g. If 3D-printed from PLA, this drops to 5.6 g. The SPINE-type base alone contributes 3.5g to the total mass.

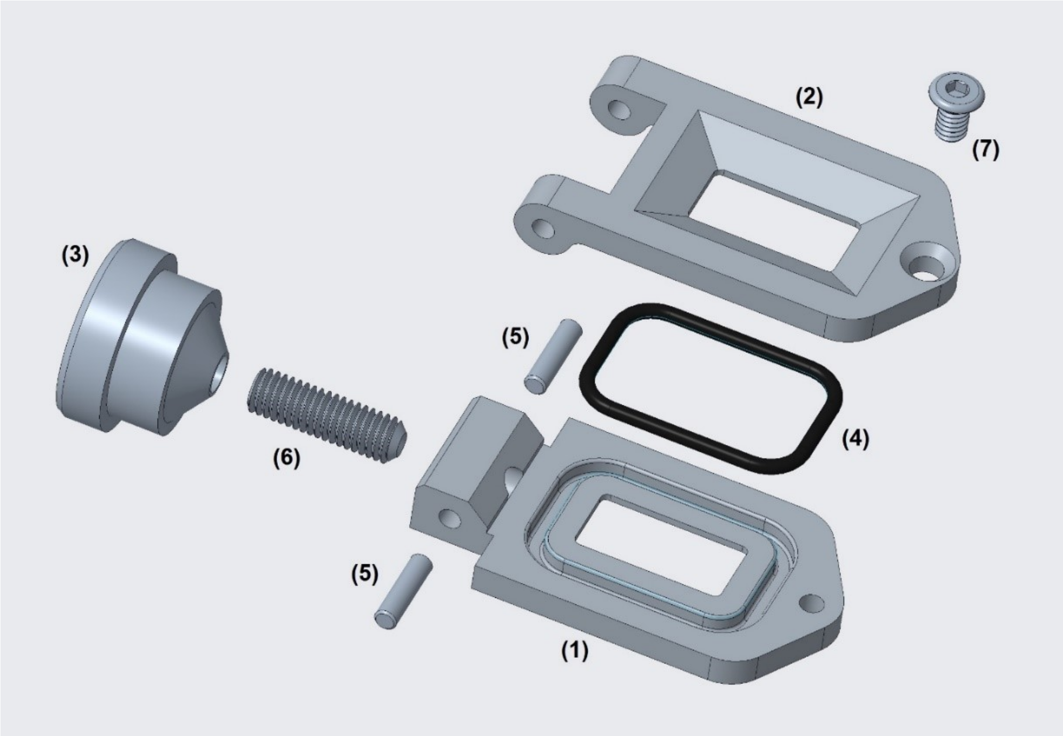

Figure S1 Exploded view of the SOSOS chip with its constituent components numbered.

Table S1 SOSOS components as numbered in **Figure S1**.

#	Item	Description	Remarks
1	Base Frame	Fabricate	Al, PEEK, PLA, etc.
2	Hinged Cover	Fabricate	Al, PEEK, PLA, etc.
3	Goniometer Button	CP111-014N	Crystal Positioning Systems
4	O-ring	M13x1	Viton or Buna N, Durometer 70A to 75A
5	Dowel Pin	M1.5-6	Stainless steel
6	Set Screw	M3x0.5-10	Stainless Steel
7	Flat-Head Screw	M2x0.4-4 to -6	Stainless Steel, length 4 to 6 mm

The clamping screw is a standard 2 mm stainless steel metric screw (thread pitch of 0.4 mm). Particularly if the base frame and cover are 3D-printed from plastic, it is easy to strip the through-threaded hole in the base plate by overtightening this screw. The use of a Torx-head or Allen-head screw is recommended, in conjunction with a precision-control swivelling screwdriver to mitigate over-tightening. Stainless steel helical thread inserts are available in the M2x0.4 size, along with the

corresponding tap-drill and insertion tool. These inserts can be installed either during the initial fabrication to strengthen the threads or later to repair a damaged thread.

S1.1. Sample spreading

Particularly for viscous samples, but often also for aqueous samples, a simple hand press is useful for pressuring the sample into a thin uniform layer. Such a press for the SOSOS chip is shown in **Figure S2**. The active pressure face was fabricated from a microscope slide, cut to shape to just fit within the 5 mm x 10 mm window of the SOSOS window. The edges of the glass plate were bevelled to avoid cutting the films while pressing, and the plate was attached to a standard optical post by means of double-sided foam tape, 1/16" inch thick. Care must be taken not to apply so much pressure as to damage the crystals. By letting the weight of the assembly alone apply the force, a consistent force can be applied every time. By varying the number, diameter, and length of the chosen posts, the pressure on the SOSOS films can then be adjusted as needed.

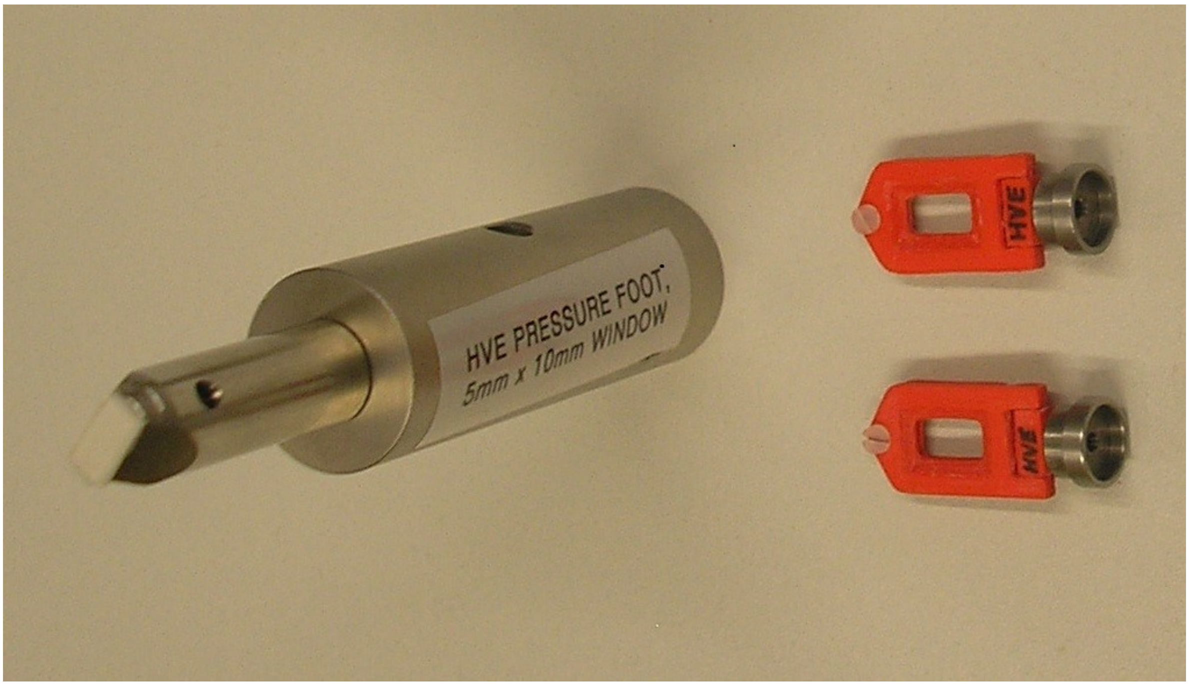

Figure S2 Simple pressure foot employed to help form viscous sample between the two constrain films into a thin uniform layer. Also shown a two SOSOS units 3D printed in PLA using the attached STP drawings.

S1.2. Orienting the SOSOS chip with respect to the X-ray beam direction

The chip surface should be exactly orthogonal to the X-ray beam. This is necessary for efficient data collection, as well as for efficient indexing of the diffraction patterns, as it maintains an identical

sample-to-detector distance for all points on the chip. When mounted on the usual goniometer button, which is not keyed to a fixed azimuthal orientation, this orthogonality must be set manually every time the SOSOS chip is mounted by (i) focusing the monitoring microscope on the center of the window; (ii) translating the film transversely to bring the edge of the sample region into view; (iii) rotating the goniometer head about its axis until the film at this edge comes into focus; and finally, (iv) checking by translating to the opposite edge of the sample region and verifying that the film is still in focus.

For an endstation that does provide a fixed azimuthal orientation, an adaptor can be designed that mechanically keys to the fixed orientation and carries it through to the SOSOS chip. An example is shown in **Figure S3**. Alternatively the SPINE goniometer base can simply be removed from the SOSOS chip and M3 screw hole in the SOSOS frame employed to attach the chip to the proprietary base, as was done at ID29 (Grieco *et al.*, 2024, Stubbs *et al.*, 2024). The chip must be unscrewed from the proprietary base for loading.

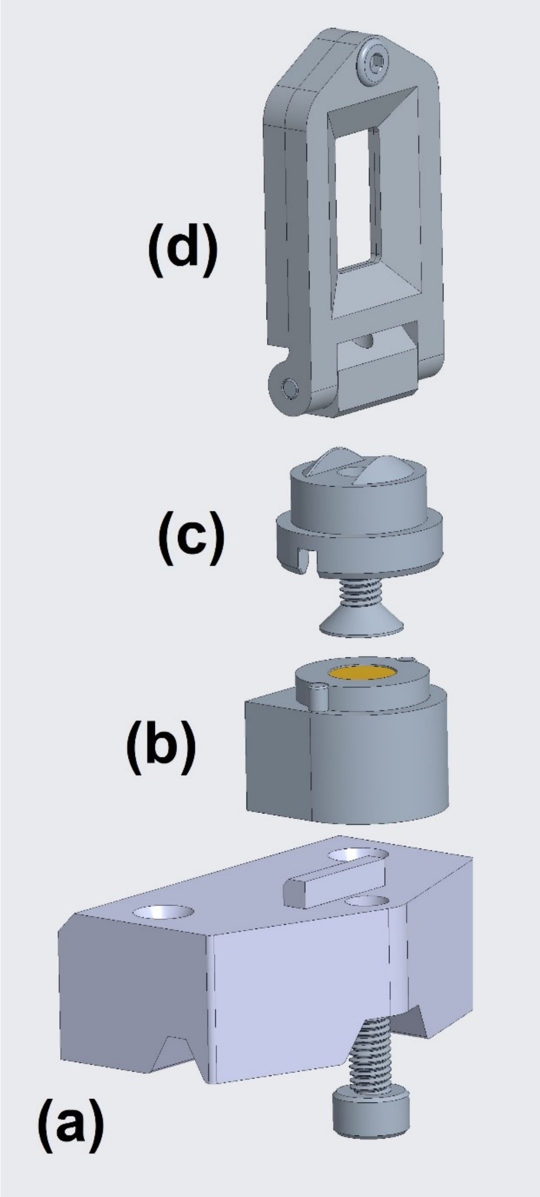

Figure S3 Possible method of orienting the SOSOS films perpendicular to the X-ray beam direction while still retaining the rapid magnetic attachment of a SPINE-type connection. A proprietary mounting base (a) self-orients with respect to the X-ray beam. A slot in a custom-designed pedestal (b) carries this orientation upward by fitting tightly over a raised boss on the mounting base. Two dowel pins on the upper side of the pedestal key into slots machined into the SPINE-type button of the SOSOS assembly (c), and this orientation is then transferred to the SOS frame by a close-fit notch machined into the top of the button.

SOS Chip

The constituent components of the basic SOS chip are shown and numbered in the exploded view of **Figure S4**. Details are provided in the following **Supplementary Table 2**. The through-hole in the base plate is 30 mm x 30 mm. That in the cover plate is 35 mm x 35 mm. With the base plate and cover fabricated from aluminium, the mass of the SOS assembly shown in **Figure 4** is 22.3 g. If the base plate and cover are 3D-printed from PLA at 100% fill, this drops to 11.8 g. The mass of the chip is generally much less than that of the mounting brackets that attach it to the goniometer, such as those seen below the SOS chip in the photograph of **Figure 1**.

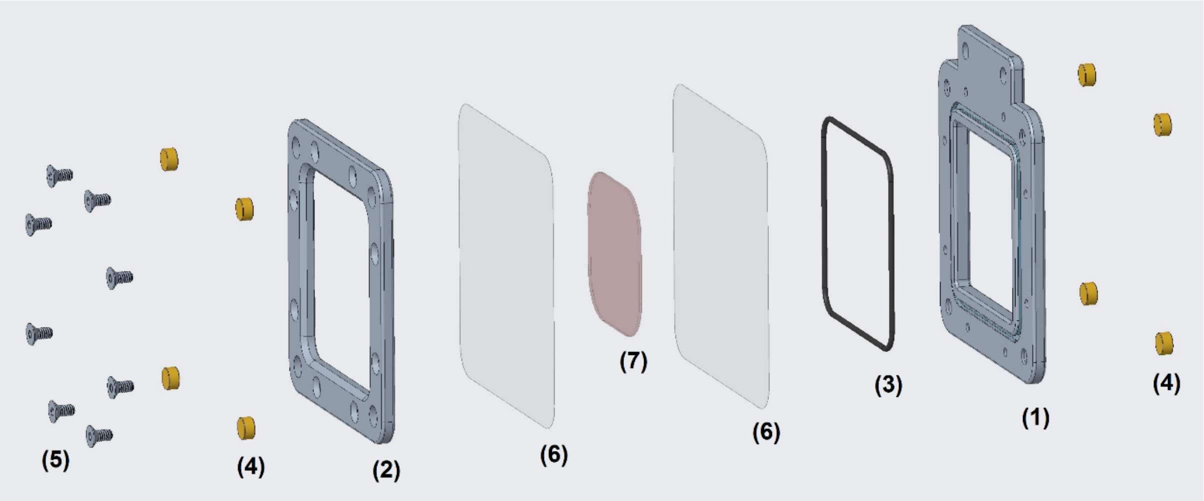

Figure S4 Exploded view of the SOS chip, with constituent components numbered.

Table S2 SOS components as numbered in **Figure S4**.

#	Item	Description	Remarks
1	Base Plate	Fabricate	Al, PEEK, PLA, etc,
2	Cover Plate	Fabricate	Al, PEEK, PLA, etc.
3	O-ring	M42x1	Viton or Buna-N, Durometer 70A to 75A
4	Magnets	Nd, 4 mm OD x 2 mm thick	13.3-13.7 kG
5	Flat-Head Screws	M2x0.4-5	Stainless Steel
6	Polymer Film	Typically 2 to 6 µm thick	Assorted polymer materials
7	Sample	Slurry of microscopic crystals	Spread on lower film during loading

Small disk magnets are not generally marked as to polarity. During fabrication it is therefore important to physically verify the correct orientation of each magnet before it is permanently affixed in place. Magnets in the base plate must be attracted to those in the loading plate and those in the chip mount and magnets in the cover must be attracted to those in the base plate. For universality of the magnetic mounting, this must be true for all chips relative to all magnetic mounts and all loading plates.

S1.3. Thinning and spreading the sample layer

Particularly for viscous samples, but often also for aqueous samples, a simple press is useful for pressing the sample into a thin uniform layer. Such a press for the SOS chip is shown in **Figure S5**. The press shown in the photo of **Figure S5** was constructed entirely from standard optical components. A thumb-knob-driven linear drive provides the press motion. It is bolted to a standard optical bread board and an optical post is attached to the linear drive with a right-angle bracket. On the bottom of the optical is the pressure foot, cut from a microscope slide to just fit into the SOS window. The edges of the slide are slightly rounded to avoid cutting into the films. The glass slide is attached to the drive rod with 1/16" thick double-sided foam tape to allow a small amount of angular auto-adjustment. Before use, the face of the microscope slide is adjusted to be parallel to the face of the loading plate by lowering the glass face to contact the optical plate, pressing moderately hard, then loosening and retightening the four attachment screws of the right-angle bracket. The alignment can be checked by placing a short strip of paper under each corner, applying slight pressure on the paper strips with the press, and tugging on the strips to see if all four are gripped with approximately the same force. We have since constructed much more sophisticated presses, but the simple version of **Figure S5** works fine.

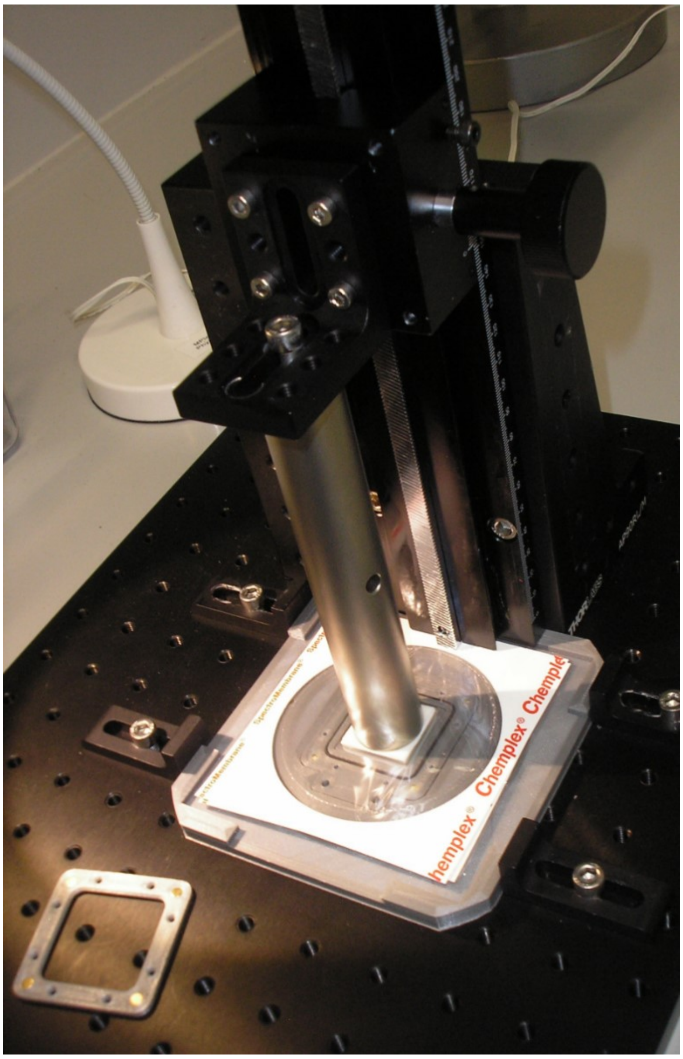

Figure S5 After the SOS sandwich of film-sample-film has been formed, a simple press can be employed to pressure the sample into a thin, uniform layer before the 8 clamping screws are tightened. This is generally necessary when working with high viscosity sample slurries, but can also help to thin ad spread aqueous sample slurries.

S2. CAD Files for SOSOS and SOS Components

To facilitate fabrication of SOSOS and SOS chips for research and education purposes, STP files for the critical components are provided in **Table S3**. The STP files in the left-hand column of the table may be copied directly from the table and pasted into the relevant CAD directory.

We prefer to machine the chip frames from aluminium, as indicated in the comments of **Table S3**. Nonetheless, we have also been successfully 3D-printed them from PLA on an additive 3D printer of only moderate resolution (0.4 mm lateral by 0.06 mm vertical). Given the complex shape of the form-fitted recesses in the loading plates, 3D-printing is the logical choice in that case. 3D additive printing invariably delivers slightly off-size dimensions (inner diameters slightly smaller than specified; outer diameters slightly larger). The amount depends on the printer, printing parameters, and material. Machined components, in contrast, are cut to the dimensions specified in the drawings. In the STP files for the loading plates, this off-sizing has been taken into account for our specific printer. With other printers, it may be necessary to adjust the dimensions slightly to get a snug fit.

These drawings may be used under the usual free-use/fair-use conventions for education and research. R. Bruce Doak and the Max-Planck-Gesellschaft retain any and all copyrights and intellectual property rights inherent to the drawings.

Table S3 STEP files for critical components of SOSOS and SOS chips.

STEP File	File Size, Reference	Comments
SOSOS_5x10mm_BASE_Al.stp	133 kB<br>Fig. S1 (1)	Part was machined from Al
SOSOS_5x10mm_COVER_Al.stp	99 kB<br>Fig. S1 (2)	Part was machined from Al<br>See Note (1)
SOS_30x30mm_LOADING_PLATE.stp	138 kB<br>Fig.6, (a)	Part was 3D-printed from PLA
SOS_30x30mm_FRONT_FRAME_Al.stp	230 kB<br>Fig. S4, (1)	Part was machined from Al;<br>See Note (2)
SOS_30x30mm_REAR_FRAME_Al.stp	127 kB<br>Fig. S4 (2)	Part was machined from Al
SOSOS_5x10mm_LOADING_PLATE_PLA.stp	80 kB<br>Fig. 3, (a)	Part was 3D-printed from PLA<br>See Note (1)
SOS_30x30mm_GENERIC_MAG_CRADLE.stp	100 kB<br>Fig, 1, right, cradling SOS	Part was machined from Al<br>See Note (3)

Note (1) Additive 3D printing leaves a slight ripple in printed surfaces, even in planar surfaces orthogonal to the print-nozzle axis. This ripple can be impressed onto the sample layer during loading, producing ripples in thickness of the sample layer. It is therefore recommended, after

printing, to grind or sand completely smooth and flat those surfaces of the loading plate that will be in direct contact with the films that have the sample sandwiched between them.

Note (2) The O-ring groove in the drawing is shown with a slanted inner wall to capture the O-ring. In the machined aluminum SOS frame it was found that a parallel-sided O-ring grove of 1.0 mm width would also retain the O-ring (inner wall of the O-ring groove 35.0 mm x 35.0 mm). When the chips are to be stored for any length of time, the clamping screws should be loosened by about one rotation to reduce flattening of the O-ring. It is important to grease the O-ring before insertion into the O-ring groove and again as needed thereafter.

Note (3) Two 5 mm diameter x 2 mm thick disk magnets are press-fitted or glued into the corresponding blind holes of the SOS generic magnet cradle. The proper orientation of the magnets should be verified before they are fixed in place: They must pull the SOS frame into the recess in the mounting cradle when the chip is oriented as in the right panel of **Figure 1**.

S3. Damage spreading due to radicals, dehydration, etc.

To protect against crystal dehydration and ultimately sample desiccation, any type of chip is generally sealed inside two thin polymer sheets. A wide variety of film materials and thicknesses is readily available for this. The chosen material must prevent dehydration of the sample over the duration of the measurement while not introducing detrimentally high X-ray background due to scattering from the film. Sample desiccation is particularly problematic in XFEL measurements, given that each XFEL pulse punches an open hole through the entire SOS sandwich, leaving an open path for water evaporation. A desiccated region then forms around the hole and grows in diameter with time. Radicals or ions created by the X-ray pulse will also diffuse outwards, producing regions of damage that grow in size with time. Since raster scans back and forth across the chip necessarily return to the proximity of previous exposures, such spreading damage often dictates the allowable spacing of X-ray exposures both within and between line scans. This is depicted schematically in **Figure S6** for the idealized case of damage spreading isotropically at constant radial speed. Along the line scan itself the raster steps have been chosen to vastly outpace the spread of damage, yet even in the undemanding case of N=8, damage spreading from exposure in the previous line scan is seen to be encountered about half-way through the return scan, meaning that Δx would have be increased substantially in to avoid sampling damage in this depicted instance. The diagram ignores the fact that the damage front weakens as it spreads radially, making the situation less dire that appears in this overly simplistic diagram. But then again, in an actual scan N might easily be several hundred. It is clear that that SOS measurements should be carefully checked for the presence of radiation damage and the scan parameters adjusted accordingly.

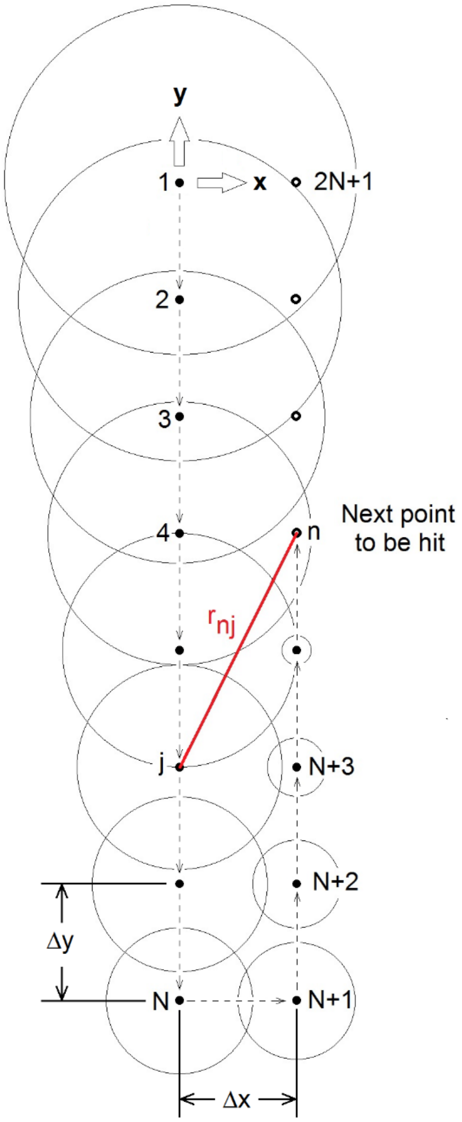

Figure S6 Schematic diagram illustrating radiation damage spreading in a step-by-step periodic exposure of a SOS chip. Shown are one full downwards scan of exposures 1 to N in steps of Δy, followed by a horizontal step of Δx = Δy and the few first exposures of the upwards return scan from N+1 to 2N+1. The damages is shown as spreading at constant radial speed. The increment Δy and the time between exposure are chosen such that damage spreads by only Δy/10 in the time between exposures.

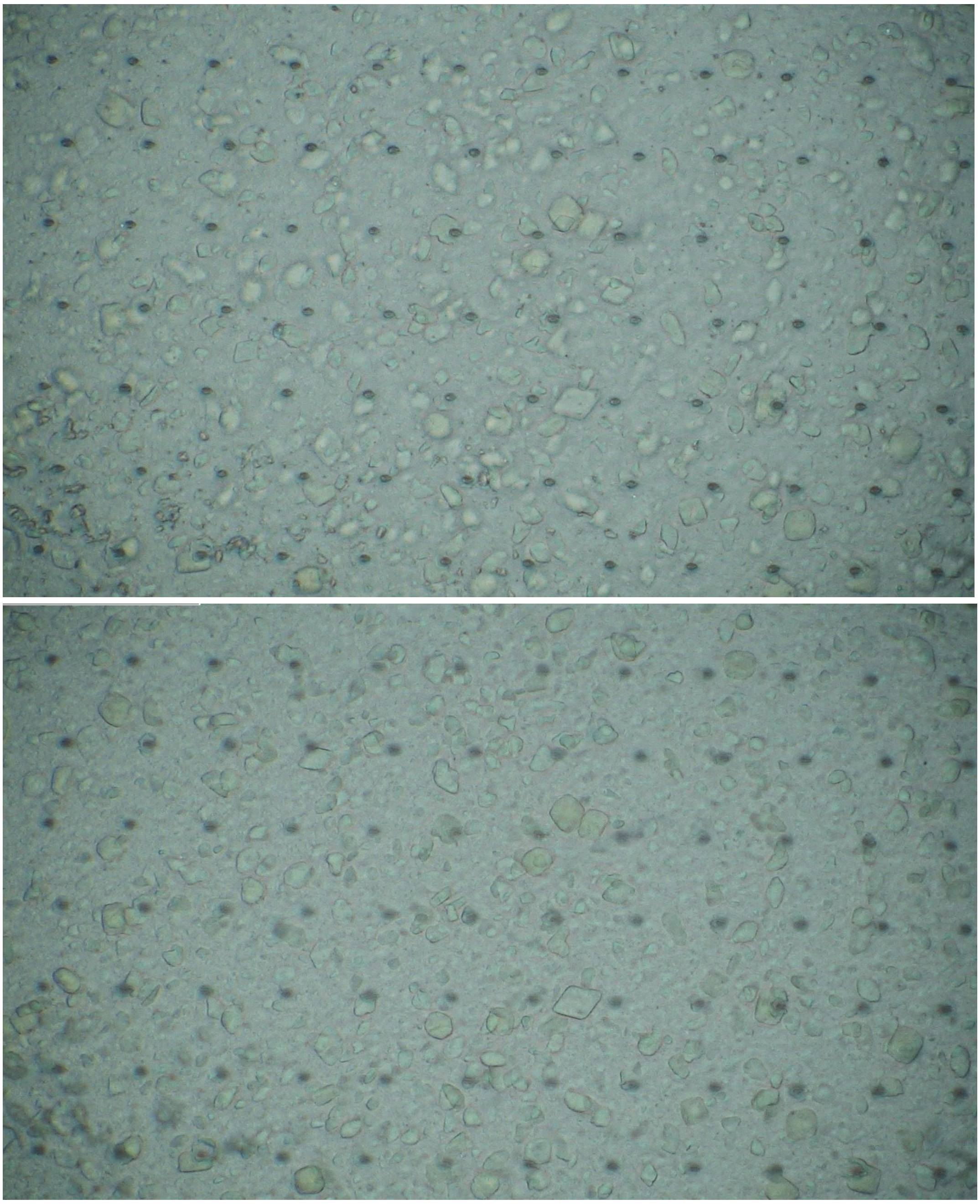

Figure S7 Micrographs of an SOS sample after data collection at Cristallina-MX at SwissFEL. The spacing between the XFEL-induced holes (dark spots) is 50 µm (horizontal and vertical). The upper picture shows the sample with the microscope focused on the inner surface of the upper Mylar sheet (thickness 6 µm), the bottom picture shows the same view with focus on the lower Mylar sheet (thickness 6 µm). The crystal thickness is ≤ 15 µm, the sample thickness about 25 ±5 µm. The sample is FAP microcrystals (Sorigue *et al.*, 2021) embedded in LCP. The LCP matrix was chosen for unrelated pump probe experiments using high viscosity extrusion for crystal delivery.

Table S4 Data collection and processing

Values for the outer shell are given in parentheses.

Diffraction source	ESRF ID-29
Wavelength (Å)	1.073 (11.56 keV, 1 % band width)
Temperature (K)	293
Detector	Jungfrau 4M
Crystal-detector distance (mm)	126
Space group	$P4_12_12$
a, *b*, *c* (Å)	57.9, 57.9, 150.3
α, β, γ (°)	90, 90, 90
Number of exposures	500,000
Number of hits	367,932
Number of indexed lattices	186,732
Resolution range (Å)	38-1.7 (1.8-1.7)
Total No. of reflections	131,935,105 (1,683,916)
No. of unique reflections	29,237 (1,412)
Completeness (%)	100 (100)
Redundancy	4474.5 (1192.6)
$\langle I/\sigma(I)\rangle$	15.1 (1.7)
R_{split}	0.067 (0.73)
Overall *B* factor from Wilson plot (Å^2) ‡	25.1

‡ The Wilson plot contains a spike at 2.2 Å due to salt spots in some images.

Thaumatin microcrystals were grown as described previously (Nass *et al.*, 2016) using seeding approaches (Shoeman *et al.*, 2023). The bipyramidially shaped microcrystals (longest dimension ca 10 µm) were suspended in mother liquor (0.8 M Na,K tartrate, 0.1 M HEPES pH 7.0) supplemented with 1.5 % (w/v) hydroxyethyl cellulose. The microcrystalline slurry was mounted in an SOS chip between two 2.5 µm thick Etnom foils using the procedure described in the main text. The ring current was ca 200 mA, X-ray transmission 40 %. The X-ray focal spot size was 2 × 4 µm. The flux was 1.5 10^15 ph/s. The beam repetition rate was 925 hz, but the acquisition rate was 231.25 Hz (so only 1 shot out of 4 was recorded), thus the exposure time was 90 µs. The average dose of the exposed region (taking into account 0.8 M Na^+/K^+ tartrate) is 1.57 MGy (Dickerson *et al.*, 2024). SX data were collected using a horizontal translational spacing of 14 µm between X-ray exposures within a line and a vertical spacing of 19 µm between scan lines. The resolution of the diffraction data is limited by the geometry

of the experimental setup. Diffraction data were analysed usig CrystFEL 0.10.0, using peakfinder8 for peak finding, xgandalf and mosflm for indexing (White *et al.*, 2012).

Table S5 Structure solution and refinement

Values for the outer shell are given in parentheses.

Resolution range (Å)	38-1.7 (1.73-1.7)
σ cutoff	none applied
No. of reflections, working set	2825
No. of reflections, test set	1412
Final R_{cryst}	0.158
Final R_{free}	0.169
Cruickshank DPI	0.072 Å
No. of non-H atoms	
Protein	1570
Ligand	10 (tartrate)
Water	127
Total	1708
R.m.s. deviations	
Bonds (Å)	0.013
Angles (°)	1.751
Average *B* factors (Å^2)	
Protein	30.3
Ligand	27.6 (tartrate)
Water	40.3
Ramachandran plot	
Most favoured (%)	97.5
Allowed (%)	2.5

Refinement was done using REFMAC 5.8.0405 (Murshudov *et al.*, 2011) using PDB 5FGT as a starting model for molecular replacement. The structure has been deposited with the PDB (code 9G2J).
